# 3D Porous Gelatin/PVA Hydrogel as Meniscus Substitute Using Alginate Micro-Particles as Porogens

**DOI:** 10.3390/polym10040380

**Published:** 2018-04-01

**Authors:** Alessandra Marrella, Alberto Lagazzo, Elena Dellacasa, Camilla Pasquini, Elisabetta Finocchio, Fabrizio Barberis, Laura Pastorino, Paolo Giannoni, Silvia Scaglione

**Affiliations:** 1 CNR-National Research Council of Italy, IEIIT Institute, Via De Marini 6, 16149 Genoa, Italy; alessandra.marrella@ieiit.cnr.it (A.M.); camilla.pasquini12@gmail.com (C.P.); 2 Department of Experimental Medicine, University of Genoa, Largo L.B. Alberti 2, 16132 Genoa, Italy; paolo.giannoni@unige.it; 3 Department of Civil, Chemical and Environmental Engineering, University of Genova, via all’Opera Pia 15, 16145 Genoa, Italy; alberto.lagazzo@unige.it (A.L.); Elisabetta.Finocchio@unige.it (E.F.); fabrizio.barberis@unige.it (F.B.); 4 Department of Informatics, Bioengineering, Robotics and Systems Engineering, Via all’ Opera Pia 13, 16145 Genova, Italy; elena.dellacasa@edu.unige.it (E.D.); laura.pastorino@unige.it (L.P.)

**Keywords:** polyvinyl alcohol, gelatin, meniscus, ex vivo culture, porous hydrogel, alginate micro-particles, porogen leaching

## Abstract

One of the current major challenges in orthopedic surgery is the treatment of meniscal lesions. Some of the main issues include mechanical consistency of meniscal implants, besides their fixation methods and integration with the host tissues. To tackle these aspects we realized a micro-porous, gelatin/polyvinyl alcohol (PVA)-based hydrogel to approach the high percentage of water present in the native meniscal tissue, recapitulating its biomechanical features, and, at the same time, realizing a porous implant, permissive to cell infiltration and tissue integration. In particular, we adopted aerodynamically-assisted jetting technology to realize sodium alginate micro-particles with controlled dimensions to be used as porogens. The porous hydrogels were realized through freezing-thawing cycles, followed by alginate particles leaching. Composite hydrogels showed a high porosity (74%) and an open porous structure, while preserving the elasticity behavior (*E* = 0.25 MPa) and high water content, typical of PVA-based hydrogels. The ex vivo animal model validation proved that the addition of gelatin, combined with the micro-porosity of the hydrogel, enhanced implant integration with the host tissue, allowing penetration of host cells within the construct boundaries. Altogether, these results show that the combined use of a water-insoluble micro-porogen and gelatin, as a bioactive agent, allowed the realization of a porous composite PVA-based hydrogel to be envisaged as a potential meniscal substitute.

## 1. Introduction

The soft tissues of the musculoskeletal system, such as articular cartilage and meniscus, cover an important role in the efficient and pain-free execution of the activities of daily living. However, as a direct consequence of the central role of these tissues in load bearing, they are commonly injured, thus becoming a significant healthcare burden worldwide [1]. Injuries of menisci are the most common in the knee joint due to the forces they are usually subjected to, causing pain, altering and locking knee joints [2].

Menisci are composed of fibrocartilagineous tissue. They are placed between the femoral condyle and flat plateau and their main role is to increase congruency of shape between the curved condyle and flat plateau, as well as to confer joint stability and to transfer loads. In fact, menisci play basically a biomechanical role, distributing loads and contact forces at the level of articulations and absorbing and dissipating biomechanical shocks [3,4].

Unfortunately, the human meniscus, mostly in the avascular area, has a limited regenerative capability, therefore partial or total meniscectomy are the most adopted solutions for pain relief [5], but these clinical approaches often lead to degenerative changes in the joints physiology [6,7,8,9], such as the induction of flat femoral condyle surface and a reduced joint space [6].

More recently, tissue engineering (TE) has suggested promising alternative approaches for the treatment of meniscus lesions [10]. Most of them involve the use of scaffolds, which act as tunable substrates to promote the replacement of the injured tissues [11,12]. In particular specific features which condition the implant performance in vivo are: a high level of biocompatibility of the scaffold [13,14]; a suitable porosity, able to allow an efficient extracellular matrix (ECM) deposition and integration with the host tissues [13]; and proper mechanical properties, needed to provide structural similarity to the target tissues [15,16].

The ideal meniscus implant, hence, should replicate at its best the viscoelastic properties of the natural tissue and the loading conditions shouldn’t affect its integrity, even after a long-term use. Moreover, the mechanical stability over time should be positively conditioned by implant integration within the host tissue. At present, there is only one FDA-approved cell-free substitute material to be used in meniscal repair; it is a collagen-based scaffold that allows cell infiltration, as reported in the literature [17]. However scaffold deterioration, degradation, and shrinkage were also shown to occur, leading to impaired functionality of the construct, ultimately evidencing the need of preservation of its mechanical features [18]. In particular, the fast degradation of the collagen-based graft (6 months to 2 years) may have hampered its clinical outcome. In this respect, a solution for meniscal repair can be accomplished by the development of bio-hybrid hydrogels structurally similar the ECM, being hydrophilic biopolymers embedded in a high-water content matrix [19,20,21,22,23]. In particular, the design of composite substitutes with a natural component, mimicking the macromolecular-based constituents of the native ECM, and with a supporting synthetic phase ensuring a slower degradation rate and performing biomechanical proprieties, may lead to functional clinical therapies for meniscus repair.

Among the biopolymers, PVA is a synthetic, biocompatible, bio-inert, and non-carcinogenic polymer with good forming ability and manufacturing, already adopted in its hydrogel form for soft tissue replacement [24,25,26]. PVA hydrogels can be easily obtained through cryotropic treatment, consisting of repeated freezing-thawing cycles, which induce the crystallization of the PVA polymer chains up to the formation of a water insoluble gel, overcoming the use of any crosslinking chemical agent that could lead to eventual toxicity issues [27]. Furthermore, PVA hydrogels exhibit rubbery and very high elastic proprieties [28], making them interesting candidates as meniscus replacement due to their high water content and to their elastic and compressive mechanical proprieties, similar to those of the native tissue [29]. PVA hydrogels were previously applied as meniscal substitutes; hydrogels prepared with different water contents, degrees of polymerization, reinforcements with other components or different microstructures and/or porosity have been evaluated mainly in vitro [30,31,32]. Only a few studies were moved on to in vivo settings: among theses, Kobayashi et al. showed that, in a sheep defect model, the meniscal replacement resulted in more performing outcomes than the untreated tissues at early time points [33]; however, on the long run, the PVA-based implant led to significantly increased cartilage degeneration, when compared with meniscal allograft transplantation, possibly due to the inability of PVA to withstand the physiological stresses and its bio-inert behavior [34]. Indeed this failure, after one year of implantation, compared to allograft transplantation, suggests that modifications of PVA-based hydrogels are necessary to improve the clinical results. In particular, despite their biocompatibility, PVA hydrogels mostly lack cell adhesion functionalities, being intrinsically bio-inert like many synthetic hydrogels. This, in turn, hampers their chances to be colonized by host cells and tissues [35]. To overcome these drawbacks, studies reported the incorporation of bioactive molecules like chitosan [36], fibronectin [35] and gelatin [37,38]. The last one, in particular, is a non-specific derivative of the protein collagen; since it carries cell adhesion ligands, blending it with PVA hydrogels results in an increased cellularity of the generated constructs [39].

Hence, given the well-known non-toxic nature and bioactivity of gelatin, we adopted this natural polymer to enhance the biological performance of the PVA-based hydrogels. A high porosity within the gelatin/PVA composite hydrogels was also introduced in order to promote integration with host tissues and matrix generation at the interface.

Despite the variety of existing methods to realize porous scaffolds, such as 3D printing, selective laser sintering, salt-based porogen leaching, compression molding, centrifugation techniques [40,41,42,43,44], most of them are hardly adaptable to the fabrication of porous water-based materials, which eventually need to be physically crosslinked to maintain a defined porous microstructure.

Nonetheless, the use of porogens that can be leached out after hydrogel crosslinking can be a suitable method to fabricate interconnected water-based structures with controlled pore size and porosity [45]. In fact, upon setting the porogen(s) size(s), a tunable pore geometry, in the range of tens to hundreds of microns, can be obtained.

However the conventional porogens, like salt, sugar, and gelatin micro-particles [46,47,48], are water soluble, hampering their incorporation in hydrophilic polymeric matrix. For this reason, we here propose an aerodynamically-assisted jetting technology to realize water-insoluble (at neutral pH) sodium alginate micro-particles with controlled size that will act as porogens. Alginate is a biocompatible natural polymer that can be easily cross-linked by divalent cations (e.g., Ca^2+^) and that can dissolve in the presence of strong calcium-coordinating ligand-based solutions, such as those containing sodium salt of the Ethylenediaminetetraacetic acid (2,2′,2″,2′′′-(Ethane-1,2-diyldinitrilo) tetraacetic acid; EDTA) [49].

Thanks to these approaches, we aimed to generate an innovative bio-inspired hybrid porous hydrogel that could: (a) provide initial biomechanical properties similar to those of the native meniscal tissue, due to the high water content typical of the hydrogels and (b) at the same time, support tissue integration to facilitate long-term stability, due to the highly interconnected porous structure easily colonized by host cells.

## 2. Materials and Methods

### 2.1. Fabrication of Alginate Micro-Particles

Alginate (Sigma Aldrich, Saint Louis, MO, USA) micro-particles were produced using an aerodynamically-assisted jetting equipment (Nisco Encapsulation Unit VAR J30, Nisco Engineering AG, Zurich, Switzerland). To this purpose, alginate was dissolved in water to a final concentration of 2% *w*/*v* by constant mechanical stirring at 1000 rpm for 2 h. The alginate solution was first filtered through a 1.20 μm syringe filter and then extruded through a conical nozzle, having a diameter of 0.25 mm, at a flow rate of 0.2 mL/min and under 100 mbar pressure. The generated micro-droplets were collected in a 0.1 M CaCl_2_ gelling solution bath under continuous stirring. The distance from the nozzle to the gelling solution was set at 6 cm. After allowing 30 min for the completion of solidification, the micro-particles were washed 3 times in distilled water by centrifugation (1000 rpm for 5 min) and kept in water until their further use.

### 2.2. Micro-Particles Characterization

Optical microscope images of the alginate micro-particles were obtained by using an inverted IX-51 Olympus microscope, quipped with a DP70 digital camera and with a CPlan 103 objective, (Olympus Italia S.R.L, Milan, Italy) and assessed for diameter analysis by the ImageJ^®^ software (1.51i version, public domain, Java-based image processing program developed at the National Institutes of Health). Briefly, optical images were converted to binary images, and the threshold of the binary was adjusted so that only the particles to be measured were highlighted. Particles that were touching were separated by watershed segmentation. The processed images were measured using a dedicated tool of the software, which generated a table outlining the particle areas. The data was then processed by Matlab software (version 9.2, The Mathworks, Natick, MA, USA), and the diameters distribution of at least one hundred alginate particles was calculated and graphed.

### 2.3. Preparation of Hydrogels

**Figure 1:**
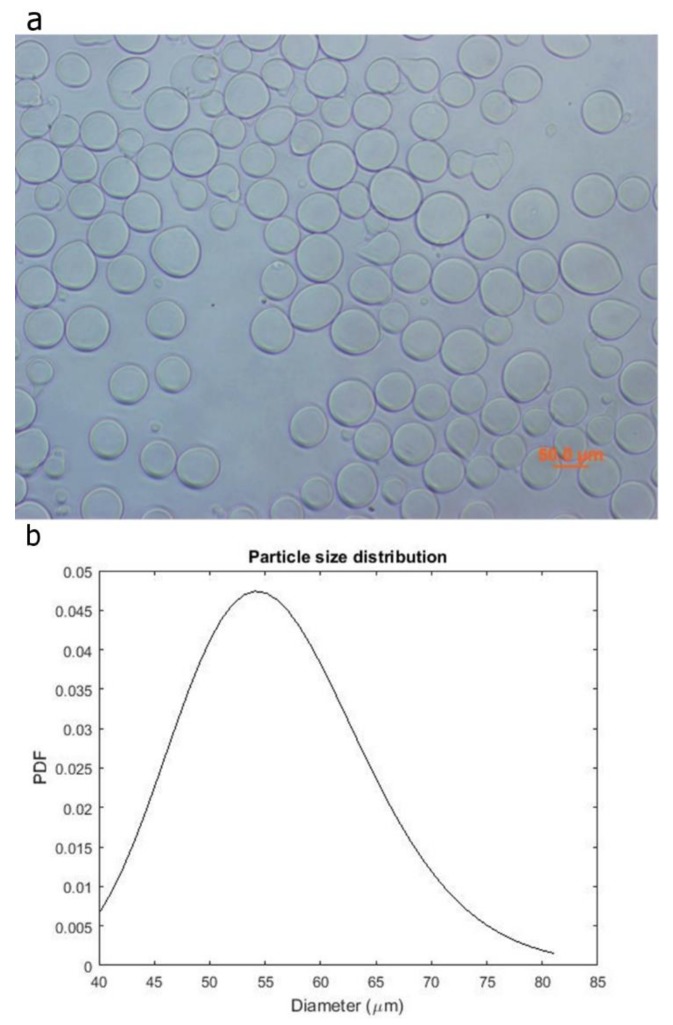
Optical image of the alginate microspheres (**a**) and the Probability Density Function of their diameter distribution (**b**).

Gelatin/PVA blend solution was prepared by dissolving 7% *w*/*v* PVA (average molecular weight 85,000–124,000 g/mol) and gelatin from bovine skin type B (Sigma Aldrich) (20/80 *w*/*w* gelatin/PVA) in deionized water at 90 °C with constant stirring for 2 h. Solutions were stirred until they cooled to room temperature to prevent the aggregation of polymers. Several ratios, between alginate and PVA/gelatin solutions were tested (data not shown), since both components may affect pore size and global porosity. A final ratio was chosen to optimize both parameters, in particular after adding alginate micro-particles (0.5 g) into 5 mm diameter and flat bottom molds, 1 mL of hybrid solution was cast drop-wise. The molds were centrifuged to allow packaging of the alginate micro-particles and air bubbles removal. Molds were then placed at −20 °C and five freezing-thawing cycles were performed to crosslink the polymeric solution (20 h at −20 °C and 4 h at 25 °C). Then, the new-formed hydrogels were immersed into 0.1 M EDTA solution at 37 °C for different incubation times (2, 5, and 10 days), to completely leach out the alginate micro-particles. The EDTA solution was renewed every 12 h. Subsequently the micro-particles were thoroughly rinsed in water for an additional day to fully remove the EDTA. PVA hydrogels without gelatin and non-porous gelatin/PVA hydrogels were also realized as controls. The fabrication process was designed to produce 3D composite porous hydrogels of 5 mm of diameter and 3 mm of height (Figure 1).

### 2.4. Morphological Analysis of Hydrogel

The morphology of the hydrogels was evaluated through histological analysis. Hydrogels were paraffin embedded, sectioned (5 micron), dewaxed, stained with methylene blue and observed with a light microscope (Nikon H550L, Nikon, Tokyo, Japan). Histological staining of alginate micro-particles was performed, as control. Post processing image analysis were carried out to evaluate the pore size distribution and total porosity of the hydrogels scaffolds through Image J^®^ software (1.48 version, public domain, Java-based image processing program developed at the National Institutes of Health). Statistical tests were carried out for at least 6 different materials, analyzing 30 measures for each sample. Mean and standard deviation are derived. Statistical significance was taken at *p* < 0.005.

### 2.5. Chemical Characterization

FT-IR skeletal spectra of dried hydrogels were recorded after dilution of the ground sample powder with KBr (FT-IR grade, Sigma Aldrich, Saint Louis, MO, USA) (1% *w*/*w*) by a Nicolet Nexus Fourier transform instrument (OMNIC™ software, DTGS detector (Thermo Electron Corporation, Waltham, MA, USA)). FT-IR analyses were performed also over PVA and gelatin powder separately, as controls.

### 2.6. Analysis of Swelling

Three samples of both porous and nonporous hydrogels were immersed in 0.01 M PBS (phosphate-buffered saline) at 37 °C. The swelling ratio was measured at different times (30 min, 1, 2 , 24 , and 48 h), until the equilibrium state of swelling was reached. At each selected time, the samples were weighted after removing the excess of solvent with an absorbent paper. Mean and standard deviation were calculated.

The swelling ratio was determined as follow:

Swelling ratio = (*m*_w_ − *m*_d_)/m_d_

where *m*_w_ and *m*_d_ are the mean weights of wet and dry sample, respectively.

### 2.7. Mechanical Characterization

The mechanical properties of the 3D swollen scaffolds were evaluated with the dynamical mechanical analysis (DMA), an adequate non-destructive tool for biomaterial characterization. The apparatus consists in a plate supporting the sample, directly connected with a mini-shaker that confers a vertical oscillation, set between 1 to 10 Hz. The sample, of cylindrical shape (5 mm in diameter and 3 mm in height), is compressed with a cylindrical plate (5 mm in diameter), linked with a force transducer, while resting on a support plate. Exactness of the position of the specimen along the vertical axis was granted by the use of a top compressive plate with dimensions identical to those of the sample (same diameter). The displacement is measured by a laser vibrometer, focused on the plate supporting the sample. All the tests were run after an initial vertical pre-deformation equal to 10%. The measurements were repeated 4 times and the mean values and the standard deviation were evaluated.

In order to compare the mechanical features of the hydrogels with the native menisci ones, the mechanical properties of native bovine menisci were evaluated both in compression and in indentation, in the range 1–10 Hz at a penetration depth of 0.5 mm (pre-strain of 10%). Uniaxial compressive perpendicular test was performed on cylindrical samples of 5 mm in diameter obtained by cutting the meniscus with a histological punch perpendicularly to the tibial plateau in each anatomical region of the meniscus (anterior, central and posterior). To obtain parallel surfaces the cylinders were cut transversally by a sharpened blade. Indentation test was executed on sagittal slices of 5 mm in thickness, produced by cutting the meniscus with a lancet, once more in each of the meniscus regions. The indentation was performed with a spherical indenter of 1 mm in diameter in 4 different points, corresponding to the region of meniscus without vascularization, red and white zone, respectively.

### 2.8. Cell Culture

A mouse fibroblast cell line (NIH-3T3) was expanded in a Dulbecco’s Modified Eagle’s Medium (DMEM) supplemented with 10% fetal bovine serum (FBS) and 1% *v*/*v* penicillin/streptomycin. The culture media was changed twice a week. At confluence, 3T3 cells were enzymatically detached with trypsin (0.05% in phosphate-buffered saline, PBS) and counted.

Firstly, NIH-3T3 fibroblasts were seeded on gelatin/PVA films, using PVA films, as controls. Briefly, the polymeric solutions—obtained with the same procedure to realize the 3D hydrogels—were poured in 24-well plates, and 5 freezing-thawing cycles were performed to crosslink the polymers. Cells were seeded (100,000 per well) and cultured for 24 h. Cell viability was evaluated through a live/dead assay (Sigma Aldrich). Briefly, samples were washed with PBS and incubated in 2 mM calcein AM and in 4 mM EthD-1 in PBS for 15 min at 37 °C in the dark, to detect live and dead cells, respectively. Cells were then rinsed in PBS again. Positivity to either staining solution was observed by means of fluorescence microscopy (Nikon H550L, Nikon, Tokyo, Japan).

NIH-3T3s were also seeded on 3D porous gelatin/PVA hydrogels, using porous PVA hydrogels as controls. In particular, 2 millions of cells were seeded for each hydrogel. After 24 h cell-laden hydrogels were processed for histological analysis. Generated constructs were fixed in 4% buffered formalin for 2 h, then paraffin embedded, sectioned (5 mm) at different levels and stained with DAPI fluorescent nuclear dye to observe NIH-3T3 nuclei within the hydrogels. The histological sections were observed and evaluated by means of fluorescence microscopy (Nikon H550L, Nikon, Tokyo, Japan).

### 2.9. Meniscal Integration Model System Ex Vivo

We investigated the integrative capability of the hydrogel in a meniscal organ-culture model in vitro. Bovine menisci were isolated from knee joints obtained from a local abattoir. A full thickness inner columnar meniscal defect was simulated using a biopsy punch to create a 5 mm diameter concentric core in each explant. The core was reinserted with the cell-free gelatin/PVA porous, non-porous hydrogel and the same meniscus plug (sham) to evaluate the effects of the microstructure on the integration with the meniscus external rim. Meniscus-gelatin/PVA constructs (*n* = 3 per group) were cultured for 10 days in 6-well plates and incubated in Dulbecco’s Modified Eagle’s Medium (DMEM; Invitrogen, Carlsbad, CA, USA) containing 1000 U/mL penicillin/streptomycin and fetal bovine serum at 37 °C, 5% CO_2_. The medium was replaced twice weekly.

Histological staining was used to evaluate the profile of junction between the hydrogels and the meniscal outer rim. Explants were fixed in 4% paraformaldehyde. Fixed explants were dehydrated in ethanol, infiltrated with xylene, and embedded in paraffin. Slides containing 5 μm sections of the paraffin-embedded explants were stained with Hematoxylin Eosin staining and DAPI fluorescent nuclear dye to observe the nuclei of the meniscus cells. Stained sections were observed under optical microscope (Nikon H550L, Nikon, Tokyo, Japan).

## 3. Results

### 3.1. Micro-Particles Characterization

The alginate micro-particles produced using the aerodynamically assisted jetting technique are shown in Figure 1, together with the Probability Density Function of the diameter distribution. This method produced micro-particles with uniform distribution and a mean diameter of 55 ± 9 μm. No aggregation was noticed, even after the washing steps.

### 3.2. Porous Hydrogel Morphological Characterization

**Figure 2:**
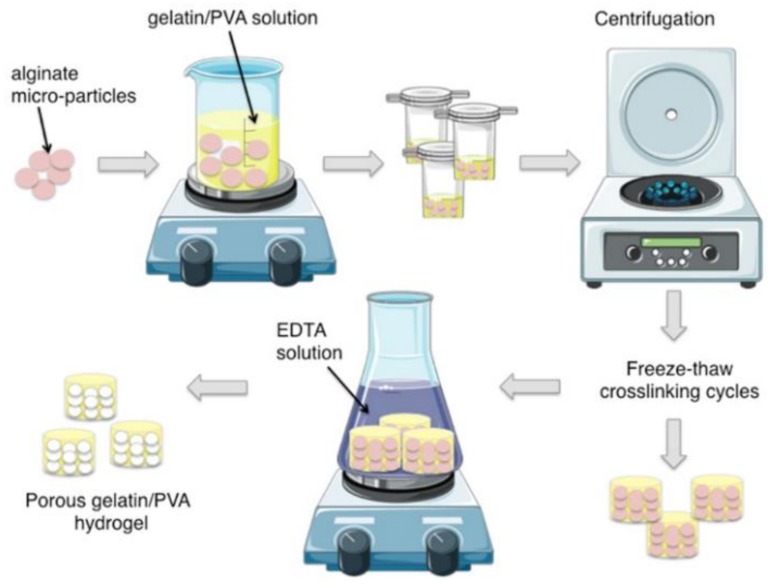
Schematic representation of the fabrication process of gelatin/polyvinyl alcohol (PVA) porous hydrogels.

Morphology of the porous hydrogel realized with the manufacturing protocol here developed (Figure 2) was assessed through histological analysis.

**Figure 3:**
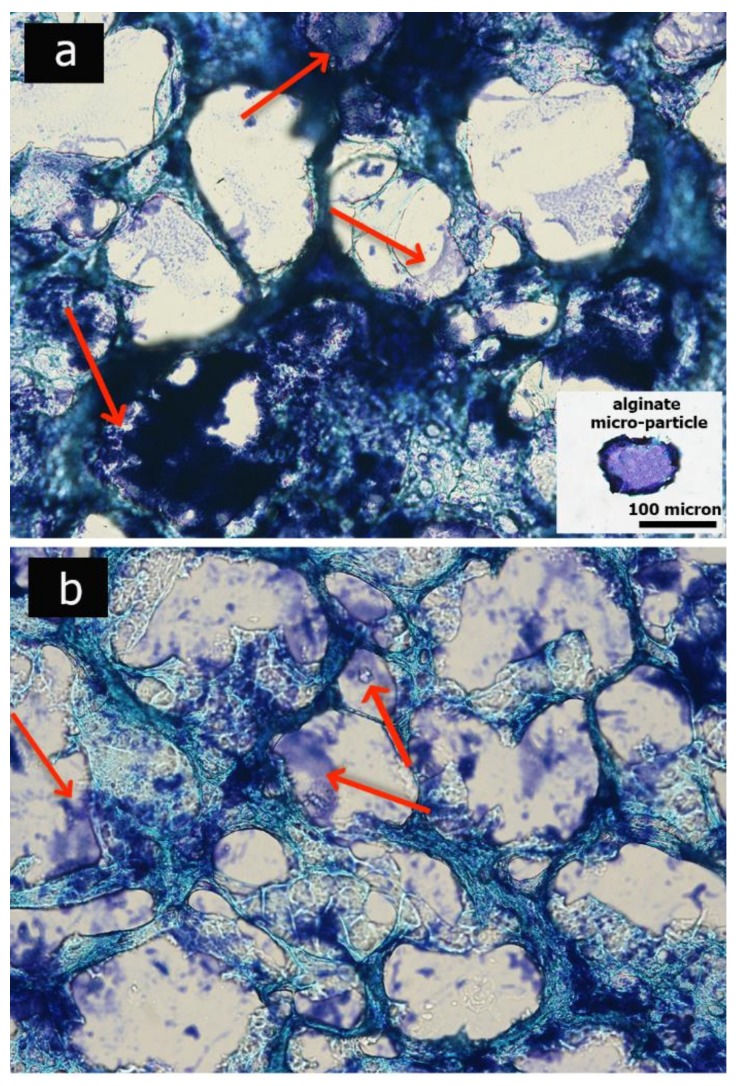

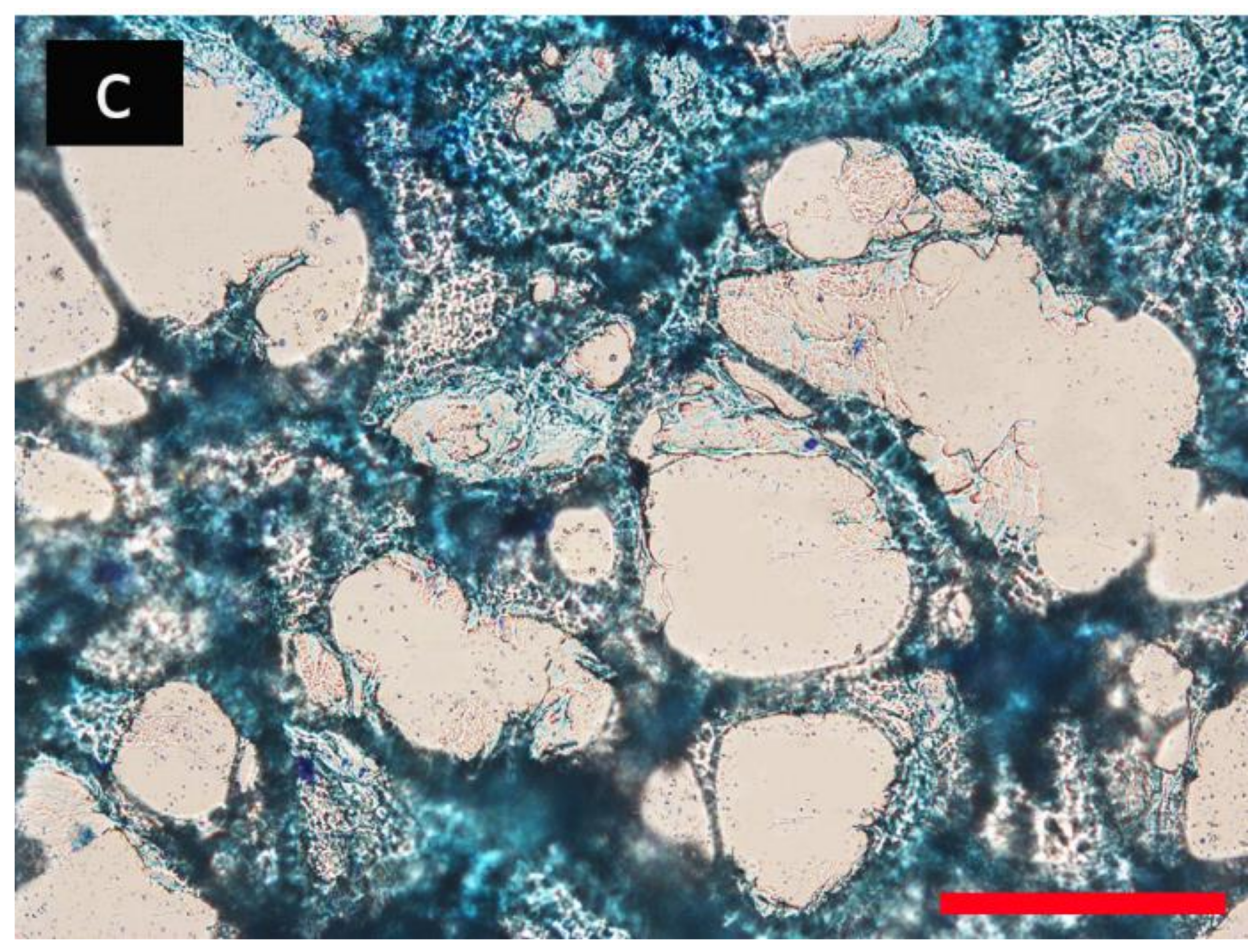
Optical images depict the micro-porosity of the hydrogels after 2 days (**a**), 5 days (**b**) and 10 days (**c**) of incubation in Ethylenediaminetetraacetic acid (2,2′,2″,2′′′-(Ethane-1,2-diyldinitrilo) tetraacetic acid; EDTA) solution. Red arrows indicate alginate residuals. Scale bar: 100 micron.

Sections were stained with methylene blue, which stains both the PVA-based hydrogels and the remaining alginate micro-particles. As shown in Figure 3, the alginate micro-particles were not completely dissolved after 2 and 5 days of hydrogel incubation in the EDTA solution, while complete dissolution was confirmed after 10 days. The final scaffold morphology appears in an inter-connected highly-porous configuration; through a semi-automatic image post-processing analysis, the mean porosity and pore size were measured, revealing a porosity of 74.5 ± 15.9% and a mean pore diameter of 104.5 ± 15.9 μm.

### 3.3. Chemical Characterization

**Figure 4:**
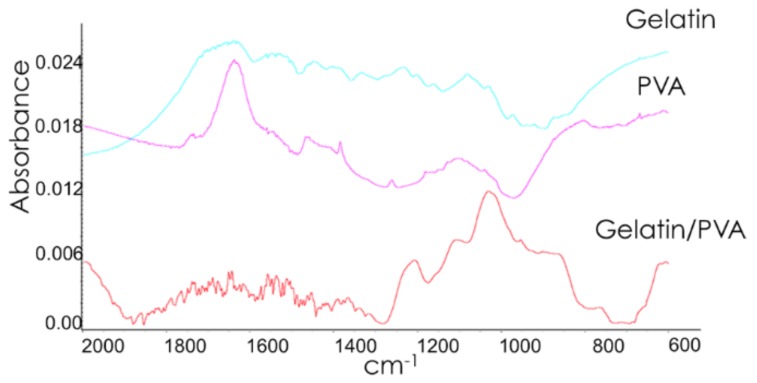
FT-IR spectra of PVA, gelatin and composite gelatin/PVA hydrogels.

FT-IR analyses were performed to investigate the absorption bands spectra of PVA, gelatin, and gelatin/PVA composite hydrogels (Figure 4). In particular, in the PVA spectrum, the bands at 1418, 1378, and 1332 cm^−1^ correspond to C–H vibrations, while the bands at 1095 and 1143 cm^−1^ are representative of CO and CC stretching vibrations [50,51].

In the gelatin spectrum, the peaks at 1560 and 1638 cm^−1^ are representative of the amide groups [37,52]. In the FT-IR spectrum of the Gelatin/PVA scaffolds, the significant peaks in the region 1600–1300 cm^−1^ are weak and considerably disturbed. In the region between 1200–800 cm^−1^, typical of CC and CO stretching vibrational modes, the spectrum shows notable differences with respect to the ones of the PVA and of the Gelatin. This is suggestive of a specific interactions, as hydrogen bonds formed between the OH groups of the polymers, thus leading to a modification of the exposed functional groups.

### 3.4. Swelling

**Figure 5:**
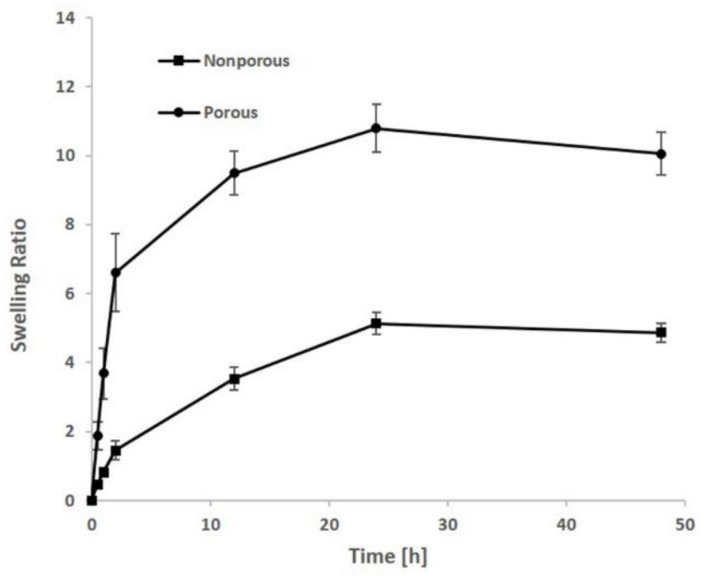
Swelling ratio of both porous and non-porous hydrogels after various soaking time in phosphate-buffered saline (PBS)-based solution.

To determine the possible influence of the porosity in the water uptake ability of the constructs, the water absorption of porous and non-porous gelatin/PVA hydrogels was measured. As shown in Figure 5, both groups showed a similar trend, and maximum swelling was reached after 24 h in PBS buffer. The swelling property of the hydrogels increased gradually up to 48 h, when the swelling equilibrium was reached. As can be observed in Figure 5, the gelatin/PVA porous hydrogels showed a good swelling behavior, being the water absorbing capacity of porous hydrogels ten times higher than their own weight. On the contrary, the absorbing capacity of the non-porous constructs was found to be only five times higher.

### 3.5. Mechanical Properties

To evaluate the effects due to porosity and to gelatin presence on the mechanical behavior of porous PVA (with and w/o gelatin) and of non-porous gelatin/PVA hydrogels we assessed the storage modulus and the loss factor, respectively, at physiological frequencies (1 and 10 Hz).

Results show that the porosity reduces considerably the storage modulus (*E*’) from 5.4 to 0.24 MPa for gelatin/PVA hydrogels (Table 1). In addition, the functionalization with gelatin causes a three-fold reduction of the stiffness of the scaffold. Despite this, the storage modulus of the gelatin/PVA hydrogels resembles the native meniscal one, since it is reported that the axial meniscal elastic modulus, at physiological loading rate and 9% of strain, varies from about 570 to 180 KPa depending on the anterior, central, and posterior region of the meniscus [53]. Mechanical characterization of the bovine menisci in swelling conditions, performed with the indentation technique on radial cross sections, at an indentation depth of 0.5 mm, displayed a storage modulus of 500 KPa at the physiological frequency of 1–2 Hz for the vascularized zone (outer zone), a compressive storage modulus around 40 KPa for a pre-strain of 0.9% in the central section, and 800 KPa for a pre-strain of 0.8% in the anterior region.

Furthermore, gelatin/PVA hydrogels show an increase of the dissipative component, assuming mechanical performances tending to a liquid-like. A loss factor (*Q*^−1^) equal to 0.16 is about twice as high as the one of porous PVA hydrogel without gelatin and it is very close to the one measured in bovine meniscus, in which *Q*^−1^ ranges between 0.14 for the not vascularized zone (inner zone) and 0.23 for the outer zone (meniscus red zone).

### 3.6. Cell Viability and Adhesion Analysis

**Figure 6:**
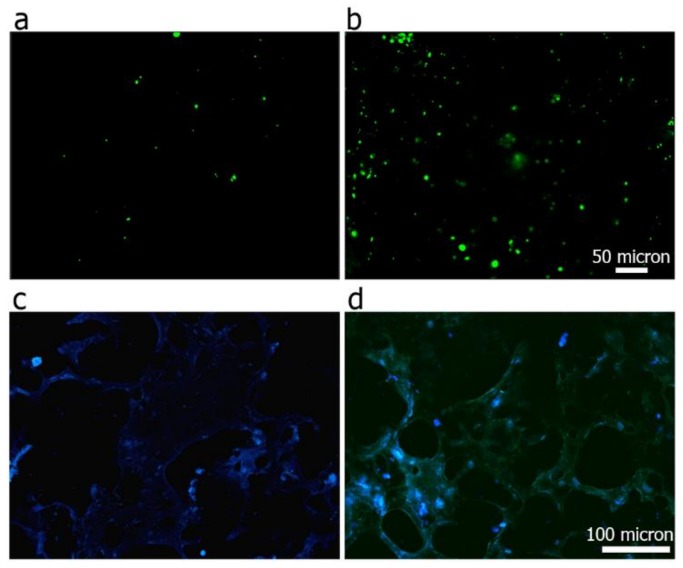
Cell viability of 3T3s after 24 h of culture on PVA (**a**) and gelatin/PVA (**b**) hydrogel film. Green spots are alive cells, red spots are dead ones. DAPI staining on histological sections representing nuclei (blue staining) of 3T3s adhering over the 3D porous PVA hydrogel (**c**), and composite porous gelatin/PVA hydrogel (**d**).

NIH-3T3 fibroblasts were seeded on the surface of the gelatin/PVA films cultured in vitro for 24 h. Dead/alive images, presented in Figure 6a,b provide clear evidence that the addition of gelatin significantly improved cell attachment. In particular, although dead cells are absent in PVA films-thus supporting the not-toxic nature of the polymer-NIH-3T3 preferred to attach on the surface functionalized with gelatin, which provided a protein component, pivotal in the process of cell adhesion.

DAPI staining (Figure 6c,d) on histological sections revealed an increased number of cells adhering on the 3D porous hydrogel surfaces functionalized with gelatin. In both cases, sections were cut along the whole height of the scaffolds, showing that they are permissive to cell penetration and colonization up to their most internal areas. Interestingly, Figure 6d showed a uniform distribution of gelatin along the scaffold, as revealed by the positive green staining, due to the auto-fluorescence of the protein.

### 3.7. Hydrogel Integration in a Ex Vivo Animal Model

**Figure 7:**
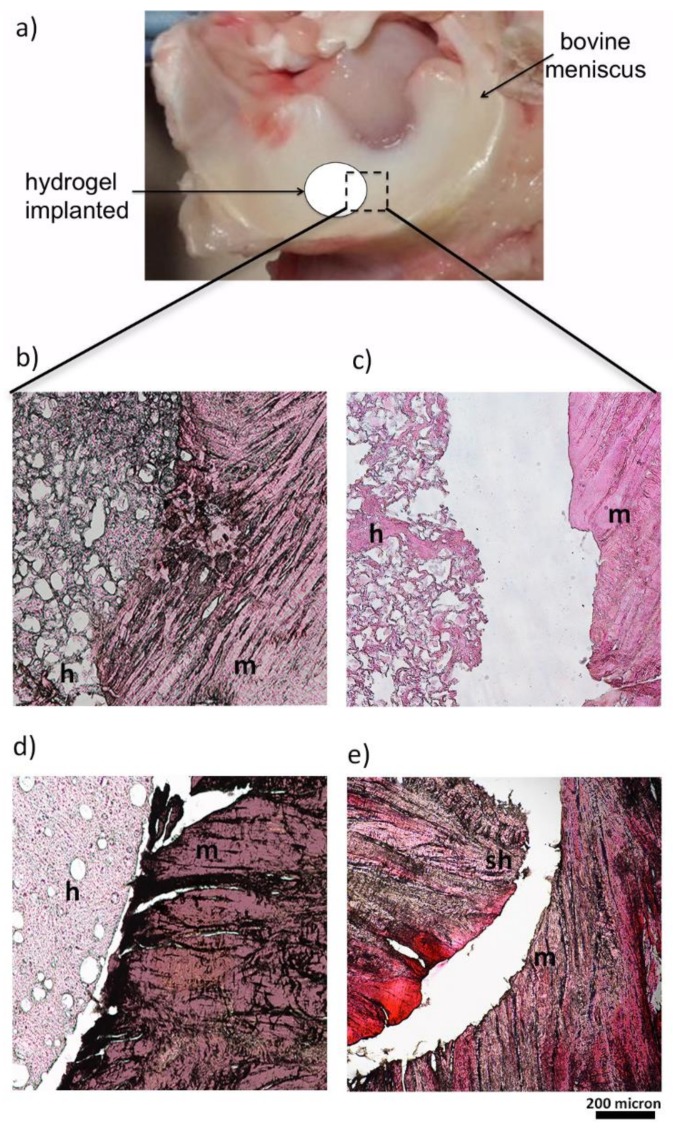
Ex Vivo culture model by bovine meniscus (**a**). Histology images (H&E) representing the junctions between the meniscal explants and the gelatin/PVA porous hydrogel (**b**), PVA porous hydrogel (**c**), non-porous gelatin/PVA hydrogel (**d**), sham (**e**) after 10 days of ex vivo culture. Meniscal explants (m), hydrogels (h), and the sham (sh) are indicated in the figure.

Although the mechanical properties of PVA hydrogels better recapitulate those of cartilaginous tissues than other hydrogels, the lack of integration between the implants and the surrounding host tissue has so far hampered their wide use in the clinics [54,55]. The capability of the hydrogels to integrate with meniscal tissue was evaluated by using an ex vivo meniscus organ-culture culture model, providing a relatively close mimicry of a clinical scenario, as already proposed for other settings [56,57]. Lesions, cast in an explanted meniscus, were repaired using the different hydrogel constructs, free of any cell components (Figure 7a); histological sections were then produced at the interface between host tissue and constructs under testing. Histological results (Figure 7b–e) evidence that the porous gelatin/PVA hydrogel was able to fit and match the surrounding meniscal native tissue, following its shape and profile. Comparison of panels 7b and 7c highlights the pivotal role of gelatin, which ensures a continuity with the protein component of the native meniscal extracellular matrix.

**Figure 8:**
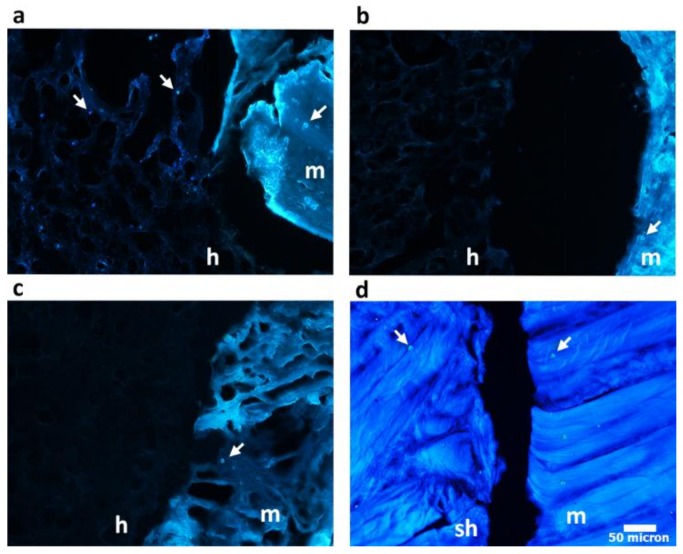
DAPI staining on histological sections representing nuclei (indicated by white arrows) of meniscus cells at the junctions level between the meniscal explants and the gelatin/PVA porous hydrogel (**a**), PVA porous hydrogel (**b**), non-porous gelatin/PVA hydrogel (**c**), sham (**d**) after 10 days of ex vivo culture. Meniscal explants (m), hydrogels (h) and the sham (sh) are indicated in the figure.

Moreover, the high porosity, combined with the addiction of gelatin in porous gelatin/PVA hydrogels, provides ground to native host cell migration, as documented by additional DAPI staining of the sections (Figure 8). Achievement of cell-free hydrogel colonization by native meniscal cells is a must to set solid bases for construct/native tissue integration, one of the major issues in the development of meniscal substitutes.

## 4. Discussion

In this study, we adopted the porogen leaching technique to fabricate an open porous hydrogel, with the aim to allow tissue infiltration, without compromising the mechanical performance ensured by the water-based nature of the grafts.

The morphological analysis of the particles (Figure 1) suggests that the proposed technique is well suited for the production of porogen micro-particles with a defined morphology. Moreover, taking into account that the micro-particles were fabricated using a commercially available droplet generator, the proposed approach is promising in the light of a scaled-up production of the developed porous scaffold.

The whole hydrogel manufacturing process (Figure 2) was finely set to ensure the formation of an open porous structure, necessary to ensure tissue infiltration and colonization. In particular, the micro-particles suspension in the polymeric solution was centrifuged to allow particle sedimentation and packaging in order to finally obtain an open porous microstructure following alginate leaching, as shown by optical images (Figure 3). After the crosslinking treatment, the excess of gel at the top of the scaffold was removed, thus obtaining a construct with micro-particles densely packed and homogeneously distributed inside the composite polymeric matrix.

During centrifugation of the micro-particles and gelatin/PVA suspension, the micro-particles underwent partial aggregation, generating pores twice bigger, in size, than the original particle mean diameter. This aspect was reproducible and the original particle size was opportunely set at about 50 micron to get a final pore size about 100 microns. The size of alginate micro-particles and the ratio between alginate and PVA/gelatin solution, affecting the pore size and global porosity of the grafts, respectively, were properly chosen as a good compromise to balance the mechanical and functional needs to promote meniscal tissue formation (data not shown). In fact, the pore size of a scaffold is an important parameter, which affects the cell differentiation and extracellular matrix production [58]. Indeed several studies proved that a porosity range of 100–200 micron is suitable to support proper cartilage formation, preventing a high degree of vascularization of the construct, which would lead towards hypertrophic cartilage [59,60,61].

Interestingly, gelatin and PVA showed specific chemical interactions and resulted in a homogeneous mixture, as shown in Figure 6d. In particular, it was possible to observe the uniform distribution of the gelatin (green autofluorescent staining) along the 3D porous hydrogels. Cell viability analysis showed that the gelatin contribution positively affected the hydrogel bioactivity, even if it underwent through the described processing steps (i.e., mixing at 90 °C and repeated freezing-thawing cycles). This was confirmed also by the histological results obtained from the ex vivo culture. Another important feature that should be taken into account during a hydrogel construct design is the swelling ratio, which is directly dependent on the hydrophilic nature and microstructure of the scaffold [62]; both swelling capacity and porosity are critical factors affecting the implant behavior in vivo [50]. An adequate water uptake capacity and open interconnected porosity allow a proper tissue infiltration and the necessary exchange of nutrients; indeed the beneficial effect of a high swelling ratio in cartilaginous matrix formation have already been proven [63]. Moreover, hydrogels with a higher swelling ratio allowed the production of more ECM components, with respect to hydrogels with lower water uptake capability [64]. Hence our novel hydrogel formulation keeps in line with these requirements, allowing to increase the construct swelling ratio, beside maintaining a sufficient mechanical stiffness, still comparable to the physiological values of meniscal cartilage.

The addition of gelatin increased the dissipative component of the hydrogel, conferring a behavior more liquid-like, and, thus, well recapitulating the high damping capability of the native meniscus, whose main role is to dissipate the physiological loads by rearranging the fluid pressurization levels within the bulk [65]. It is interesting to highlight as the mechanical proprieties of the porous PVA/gelatin hydrogels are similar to those of the native bovine menisci, both in terms of storage modulus and loss factor. This result is relevant considering the extreme structural complexity of the meniscal native tissue and, therefore, the difficulties of sampling specimens with regular geometry and relative low invasiveness of the inner structure [66]. Therefore, the combination of gelatin with a PVA-based porous hydrogels allowed to better resemble the mechanical and damping proprieties of native meniscus as well as promoting the integration with the host tissues, as shown by the ex vivo test.

## 5. Conclusions

This study describes for the first time the production and characterization of novel gelatin/PVA porous hydrogels.

Printed alginate micro-particles were used as porogens to generate a hydrogel with an interconnected porosity higher than 70% and pore size about 100 micron, uniformly distributed along the whole height of the scaffold.

The interconnected porosity enhanced the water uptake capability about five times with respect to the gelatin/PVA non-porous hydrogel, while maintaining a sufficient mechanical stiffness. In particular, the elastic modulus under dynamic compression at 1 Hz of frequency (physiologic frequency) is about 0.24 ± 0.03 MPa showing values comparable with native meniscal tissues.

The hydrogels were validated in an ex vivo organ-culture model showing that the porosity introduced through aerodynamically-assisted jetting technology improved the implants capability to fit and match to the surrounding meniscal native tissue, following its shape and profile. The idea to use a water-insoluble porogen allowed the realization of a porous structure permissive to host meniscal tissue infiltration, enhancing the chances of integration with the surrounding native tissue, without affecting the mechanical and swelling properties provided by the high water content of the PVA-based hydrogels.

## Figures and Tables

**Table 1:** Compressive storage modulus (*E*’) and loss factor (*Q*^−1^) of the hydrogels calculated at 1 Hz and 10 Hz of stimulation.

Hydrogel Sample	1 Hz		10 Hz	
	*E*’ (MPa)	*Q*^−1^	*E*’ (MPa)	*Q*^−1^
Gelatin/PVA porous	0.24 ± 0.03 *	0.16 ± 0.01	0.25 ± 0.03	0.14 ± 0.00
Gelatin/PVA not porous	5.40 ± 2.80	0.06 ± 0.01	5.44 ± 2.90	0.05 ± 0.01
PVA porous	0.78 ± 0.13	0.09 ± 0.01	0.78 ± 0.13	0.08 ± 0.02

* The reported error is the standard deviation mean calculated on four measurements.
